# Increasing Agrin Function Antagonizes Muscle Atrophy and Motor Impairment in Spinal Muscular Atrophy

**DOI:** 10.3389/fncel.2018.00017

**Published:** 2018-01-30

**Authors:** Marina Boido, Elena De Amicis, Valeria Valsecchi, Marco Trevisan, Ugo Ala, Markus A. Ruegg, Stefan Hettwer, Alessandro Vercelli

**Affiliations:** ^1^Department of Neuroscience Rita Levi Montalcini, Neuroscience Institute Cavalieri Ottolenghi, University of Turin, Turin, Italy; ^2^Department of Molecular Biotechnology and Health Sciences, University of Turin, Turin, Italy; ^3^Biozentrum, University of Basel, Basel, Switzerland; ^4^Neurotune AG, Schlieren, Switzerland; ^5^Department of Neuroscience Rita Levi Montalcini, National Institute of Neuroscience, Turin, Italy

**Keywords:** motor neuron disease, neuromuscular junctions, muscle, motor performance, innervation

## Abstract

Spinal muscular atrophy (SMA) is a pediatric genetic disease, characterized by motor neuron (MN) death, leading to progressive muscle weakness, respiratory failure, and, in the most severe cases, to death. Abnormalities at the neuromuscular junction (NMJ) have been reported in SMA, including neurofilament (NF) accumulation at presynaptic terminals, immature and smaller than normal endplates, reduced transmitter release, and, finally, muscle denervation. Here we have studied the role of agrin in SMAΔ7 mice, the experimental model of SMAII. We observed a 50% reduction in agrin expression levels in quadriceps of P10 SMA mice compared to age-matched WT controls. To counteract such condition, we treated SMA mice from birth onwards with therapeutic agrin biological NT-1654, an active splice variant of agrin retaining synaptogenic properties, which is also resistant to proteolytic cleavage by neurotrypsin. Mice were analyzed for behavior, muscle and NMJ histology, and survival. Motor behavior was significantly improved and survival was extended by treatment of SMA mice with NT-1654. At P10, H/E-stained sections of the quadriceps, a proximal muscle early involved in SMA, showed that NT-1654 treatment strongly prevented the size decrease of muscle fibers. Studies of NMJ morphology on whole-mount diaphragm preparations revealed that NT-1654-treated SMA mice had more mature NMJs and reduced NF accumulation, compared to vehicle-treated SMA mice. We conclude that increasing agrin function in SMA has beneficial outcomes on muscle fibers and NMJs as the agrin biological NT-1654 restores the crosstalk between muscle and MNs, delaying muscular atrophy, improving motor performance and extending survival.

## Introduction

Spinal muscular atrophy (SMA) is a fatal pediatric genetic disease, characterized by selective motor neuron (MN) degeneration and death, leading to progressive muscle weakness and paralysis, respiratory failure, and, in the most severe cases, to death. In humans two almost identical SMN (survival motor neuron) genes are present on chromosome 5q13. SMA is caused by mutations in the telomeric gene “survival motor neuron 1” (SMN1), that consequently cannot encode SMN protein. By contrast the centromeric SMN2 gene is not affected by the disease: physiologically SMN2 mostly generates a truncated protein lacking exon 7 (SMNΔ7), and only a limited amount of functional full length protein. SMN2 may exist in several copies and the copy number of SMN2 modulates the disease severity, depending on the amount of functional protein produced (Lorson et al., 2010). Based on the age of onset and disease severity, SMA can be classified into four main clinical types (I–IV from severe to mild) (Lefebvre et al., 1997). Currently a new therapy aimed at increasing SMN expression raised great expectations (Finkel et al., 2017), even though additional drugs are needed to counteract muscular atrophy: SMN is needed in both neurons and muscles (indeed its requirement is highest when the neuromuscular system is correctly established during development); moreover the efficacy of SMN-based treatments can be poor if the neuromuscular system is compromised, since delayed symptoms can later affect treated patients (Bowerman et al., 2017).

SMN, together with Gemins 2 to 7, is involved in the assembly of small nuclear ribonucleoprotein (snRNP) particles, and its deficiency can disrupt splicing of specific targets (Wan et al., 2005). Moreover, it has been suggested that SMN lack can result in defects in MN β-actin mRNA axonal transport, neurotransmitter release, neurofilament dynamics and accumulation at the synapse, and neuromuscular junction (NMJ) maturation (Pagliardini et al., 2000; Fan and Simard, 2002; Jablonka et al., 2004; Kariya et al., 2008; Kong et al., 2009; Torres-Benito et al., 2011). Indeed, SMAΔ7 mice, the experimental model of SMAII, recapitulate these defects, showing reduced synaptic vesicle density and concomitant synaptic transmission defects (Dale et al., 2011). Post-natal development of the NMJs is arrested in SMA, which impairs the maturation of endplate acetylcholine receptors (AChRs), determining a delay in the developmental switch from embryonic γ-subunit containing AChRs to the mature ϵ-subunit containing AChRs (Wyatt and Keirstead, 2010). Therefore, SMA murine models show abnormalities of NMJs, including neurofilament accumulation at presynaptic terminals, smaller and immature endplates, reduced transmitter release, and denervation (Ling et al., 2010).

The NMJ formation is physiologically promoted by the neurotrypsin/agrin system. Agrin, a large heparan sulfate proteoglycan, is a synaptic organizer responsible for NMJ development, i.e., insertion of a nerve terminal, clustering of AChRs and maturation of NMJs (Bolliger et al., 2010): agrin signals via the surface receptors Lrp4 and MuSK, and other signaling molecules (such as Dok-7 and rapsyn) to guarantee the efficiency and functionality of the neuromuscular transmission (Glass and Yancopoulos, 1997; Zong et al., 2012). Agrin is cleaved by neurotrypsin, a member of the serine protease family S1A, at two homologous, highly conserved sites, resulting in the release of the C-terminal domain (agrin-22) containing the NMJ-organizing and protecting function (Reif et al., 2007). Such cleavage inactivates the AChR clustering activity of agrin.

Here we have studied the role of agrin in preventing muscular atrophy in SMA as we hypothesized that stabilization of NMJs would improve the crosstalk between muscle fibers and MNs and thus delay disease progression, muscle atrophy and motor impairment. We therefore daily administered to SMA pups a C-terminal fragment of mouse agrin, termed NT-1654, which is neurotrypsin-resistant, highly soluble and retains the AChR-clustering activity. We provide evidence that this treatment is effective at the neuromuscular level, delays disease progression and significantly improves motor performance.

## Materials and methods

### Animal care and genotyping

All procedures involving animals were performed in accordance with national (DL n. 116, G.U., Supp. 40, February 18, 1992; permit number 17/2010-B, June 30, 2010) and European Community Council guidelines (Council Directive 86/609/EEC). Additionally, an ad hoc Ethical Committee of the University of Turin specifically approved this study. Particular care was taken to minimize the number of animals, their discomfort and pain.

The original breeding pairs of SMA mice were purchased from Jackson Laboratory (stock number 005025; Jackson Lab, Maine, USA). Mice had free access to food and water. The colony was maintained by interbreeding carrier mice, and the offspring were genotyped by PCR assays on tail DNA according to the protocols provided by Jackson Laboratory: briefly, animals were genotyped for the presence of the two human transgenes (SMN2 and SMNΔ7) and the three possible genotype variants of the mouse *Smn* locus (Smn+/+, Smn+/– and Smn–/–). Sequences of primers and probes (5′-3′) used for WT murine *Smn* gene are TTTTCTCCCTCTTCAGAGTGAT (forward), CTGTTTCAAGGGAGTTGTGGC (reverse). Sequences of primers and probes (5′-3′) used for mutant murine *Smn* gene are TTTTCTCCCTCTTCAGAGTGAT (forward), GGTAACGCCAGGGTTTTCC (reverse).

Data were obtained from tissues harvested from SMA (Smn−/−) and WT (Smn+/+) mice, killed at postnatal day 10 (P10), considering P0 as the day of birth. Just for western blot analysis additional animals were sacrificed at P5.

In order to reduce the number of used animals, data concerning quadriceps histological examination (hematoxylin/eosin staining and Cytochrome c Oxidase assay) on untreated pups were the same as in another paper published (Valsecchi et al., 2015).

### Agrin western blotting

Agrin expression in SMA and WT mice was measured at P5 (3 WT and 4 SMA) and at P10 (4 WT and 3 SMA). Animals were killed by cervical dislocation and their quadriceps were rapidly dissected on ice. Muscles were homogenized in liquid nitrogen and collected in ice-cold lysis buffer (20 mM Hepes pH 7.5, 1% Triton, 150 mM NaCl, 10 mM NaF, 1 mM Na_3_VO_4_, 0.5 μg/ml apronitin, 1 μg/ml leupeptin, 1 μg/ml pepstatin). The samples were then cleared by centrifugation and the supernatant was used for Western blot analysis after protein determination by the Bio-Rad protein assay (Biorad, Milan, Italy). Samples with equal protein concentration were resolved by SDS-PAGE on 4–12% NUPAGE gels using MES running buffer (Life Technologies) and subsequently blotted to nitrocellulose membrane (Ge Healthcare, Milan, Italy). To detect the proteins of interest, specific antibodies were used: anti-agrin (mouse monoclonal, low affinity anti-C22 antibody, 1.5 μg/ml), provided by Dr. Hettwer (Stephan et al., 2008), and anti-GAPDH (mouse monoclonal, 1:1,000, Millipore, Temecula, CA, USA). Immunoreaction was revealed using anti-mouse IgG conjugated to peroxidase, 1:4,000 (Ge Healthcare) by the ECL reagent (Ge Healthcare). The optical density of the bands was determined by Chemi Doc Imaging System (Biorad) and normalized to the optical density of GAPDH.

### Agrin administration

The agrin biological NT-1654 was designed (US20120208765 A1 patent number), synthesized and supplied by Neurotune AG (Schlieren, Switzerland). It consists of a 44 kDa protein containing the C-terminal part of the 220 kDa full-length agrin. It corresponds to an active splice variant of agrin that is secreted by the MNs and includes the “synapse-inducing” activity. To protect it from the cleavage by neurotrypsin, it is mutated at the cleavage site.

NT-1654 (10 mg/kg) was administered subcutaneously daily in SMA mice, starting from P0. This agrin formulation is highly soluble and the administration route is effective to reach all neuromuscular target site. Moreover, this concentration has been demonstrated to be safe and therapeutic when employed in a sarcopenia murine-model (Hettwer et al., 2014). Sham-injected pups received sterile phosphate buffer solution (PBS; pH 7.3).

For histological examination, the animals were sacrificed at P10 (NT-1654 SMA *n* = 12, PBS SMA *n* = 15). In another set of experiments, additional mice (PBS SMA *n* = 20, NT-1654 SMA *n* = 19) were used for survival analysis.

### Behavioral tests

Motor behavior and wellness of the animals were assessed every second day, starting from P2 (NT-1654 SMA *n* = 27, PBS SMA *n* = 15). Before starting the behavioral tests, the animals were weighed. According to El-Khodor et al. (2008), a battery of behavioral tests have been selected: (i) Righting reflex. Pups were placed on their backs on a flat surface. The time employed to right themselves was recorded (cut off 30 s). (ii) Tail suspension test (self-clasping). Pups were suspended by the tail for 15 s and their hindlimb posture was scored: 4, hindlimbs spread open; 3, hindlimbs not completely spread; 2, hindlimbs often close together; 1, hindlimbs always close together; 0, hindlimbs always close together with clasping. (iii) Hindlimb suspension test (tube test). The test required two consecutive trials; in each trial, the mouse was placed head down, hanging by its hindlimbs in a plastic 50 ml tube with a cotton ball cushion at the bottom to protect the animal in case of fall. We considered two parameters: the latency to fall (time) from the edge of the tube and the hindlimb score: 4, normal hindlimb separation with tail raised; 3, apparent weakness and hindlimbs close together without touching each other; 2, close hindlimbs, often touching; 1, hindlimbs always clasping with the tail raised; 0, constant clasping of the hindlimbs with the tail lowered. (iv) Negative geotaxis. P4 and older mice underwent this test, useful to evaluate motor coordination and vestibular sensitivity. The animals were placed on an inclined grid (at approximately 35° inclination) with the mouse head facing down. We measured the time needed for mice to orient themselves (cut off 60 s) and we assigned 60 s also to mice unable to complete the test.

### Spinal cord histological examination

P10 mice (NT-1654 SMA *n* = 11, PBS SMA *n* = 9) were deeply anesthetized by gaseous anesthesia (3% isoflurane vaporized in O_2_/N_2_O 50:50) and perfused transcardially with 4% buffered paraformaldehyde (PFA, pH 7.4). The spinal cord was removed, cut between L1 and L5 vertebral segments, and postfixed in 4% PFA for 2 h at 4°C. Samples were immersed overnight in a solution containing 30% sucrose in phosphate buffer (PB) 0.1 M at 4°C for cryoprotection, embedded in cryostat medium (Killik; Bio-Optica, Milan, Italy) and cut on the cryostat (HM 550; Microm) in serial transverse 20 μm thick sections, mounted directly onto 5% gelatin-coated slides and stained with cresyl violet (Sigma Aldrich, St. Louis, MO, USA) for Nissl staining.

Spinal MNs (NT-1654 SMA *n* = 5, PBS SMA *n* = 5) were counted on serial sections (1 every 600 μm), using a stereological technique, the Optical Fractionator, a computer-assisted microscope and the StereoInvestigator software (MicroBrightField, Williston, VT, USA). Cells were counted on the computer screen using an Optronics MicroFire digital camera mounted on a Nikon Eclipse E600 microscope. Only neurons in the lumbar tract with an area ≥80 μm^2^ (classified as alpha MNs) and located in a congruent position were counted.

### Quadriceps histological examination

Quadriceps muscles were collected from the same animals and processed, as previously described (Valsecchi et al., 2015). The muscles underwent different histological approaches.

#### Hematoxylin/eosin staining

We evaluated mean fiber area, quadriceps cross-sectional area, mean muscular fiber number of quadriceps muscles and minimal Feret's diameter (NT-1654 SMA *n* = 6, PBS SMA *n* = 5). Muscles were cut on the cryostat in transverse 20 μm-thick sections, mounted onto 5% gelatin-coated slides, stained with hematoxylin/eosin, dehydrated in graded ethanol baths (95–100%) and cleared in xylene. Reconstructions and analysis of the sections have been performed by Neurolucida software and the associated data analysis software NeuroExplorer (MicroBrightField).

#### Cytochrome c oxidase (COX) assay

This assay is performed to evaluate fiber type composition: to this aim, some muscles (NT-1654 SMA *n* = 4, PBS SMA *n* = 3) were cut on the cryostat in transverse 50 μm-thick sections free-floating in PBS and processed for the COX histochemistry. The reaction solution was composed of 10 ml PBS 0.1 M, 0.4 g sucrose, 10 mg DAB and 5 mg cytochrome C (Sigma Aldrich), and warmed at 37°C before incubation. Incubation with reaction solution required 2–3 h at 37°C on a tilting plate. The dark COX-stained sections were classified as oxidative fibers (type I), whereas light ones as glycolytic fibers (type II).

#### Immunofluorescence

Other sections (NT-1654 SMA *n* = 5, PBS SMA *n* = 8), longitudinally cut (20 μm-thickness) and mounted onto 5% gelatin-coated slides, were immunoreacted to evaluate NMJ denervation. Briefly, the permeabilization was performed with in PBS-TRITON 0.3% at RT on a tilting plate for 20′; then the sections were incubated with Alexafluor 555-conjugated α-bungarotoxin (BTX; 1:500; Invitrogen, Milan, Italy) at RT for 30′. To block unspecific binding of the antibody, the sections were incubated for 30 min at room temperature with 0.3% Triton X-100 and 10% normal donkey serum (Sigma-Aldrich) in PBS 1X (pH 7.4); after this, the sections were incubated with the monoclonal antibody anti-neurofilament 165 kDa 2H3 clone (mouse; 1:500; Hybridoma bank, Iowa, USA) in the same solution at 4°C overnight, and then with secondary antibody Alexa488 anti-mouse (1:200; Jackson Immunoresearch; West Grove, PA, USA) for 1h at RT, followed by 4′, 6 Diamino-2 phenyindole Dilactate (DAPI; Sigma Aldrich) in PBS 1:50 for 3′. Samples were washed and coverslips were mounted with a drop of PB 0.1M with glycerol.

A Nikon Eclipse 90i epifluorescence microscope and a Nikon DS-5Mc digital camera were used to observe and photograph the sections. For checking double staining and making 3D reconstructions, some slides were examined with a Leica TCS SP5 confocal laser scanning microscope.

### Diaphragm histological examination

A separate group of P10 animals (NT-1654 SMA *n* = 3, PBS SMA *n* = 4) was killed by decapitation to collect unfixed diaphragms for whole mount staining procedure. Diaphragms were incubated with α-bungarotoxin (BTX) Alexafluor 555-conjugated (Invitrogen) 1:500 in PBS 1X for 30′ at room temperature (RT), washed with PBS and fixed with 2% buffered PFA for 10′ at RT. Samples were then permeabilized with 1% Triton X-100 in PBS overnight (ON) at 4°C. After incubation with a neutralizing solution for 15′ at RT to block the free PFA (100 mM glycine in PBS), diaphragms were incubated with 0.2% Triton X-100 and 5% normal donkey serum (Sigma-Aldrich) in PBS, to block non-specific binding sites for at least 30 min at 4°C. Samples were then incubated with monoclonal anti-neurofilament 165 kDa 2H3 clone antibody (mouse; 1:500, Hybridoma bank) in blocking solution, overnight at 4°C. After washing for 1 h at 4°C, diaphragms were incubated with secondary Alexa488 anti-mouse antibody (1:200; Jackson Immunoresearch) for 2 h at 4°C. After washing at 4°C, samples were incubated in DAPI (Sigma Aldrich) in PBS 1:50 for 3′. Diaphragms were washed with PBS 1X, and finally coverslipped with a drop of PB 0.1M with glycerol.

To study NMJ structure and maturation, we analyzed the slices with a Leica TCS SP5 confocal laser scanning microscope (NT-1654 SMA *n* = 3, PBS SMA *n* = 4); moreover, for assessing neurofilament accumulation (NT-1654 SMA *n* = 2, PBS SMA *n* = 3), we used the isosurface module of Imaris software (Bitplane, Zurich, CH).

### NT-1654 administration in P10 WT animals

To evaluate the impact of NT-1654 administration on development in physiological conditions, we also injected the compound into WT pups (NT-1654 WT), from P2 to P10. Control WT mice received PBS (PBS WT).

The animals (NT-1654 WT *n* = 32, PBS WT *n* = 20) underwent the above-mentioned behavioral tests. Then, after the sacrifice at P10, we dissected quadriceps and diaphragm muscles. The quadriceps were processed for the histological examination, as described above (NT-1654 WT mice *n* = 8, PBS WT *n* = 6): with Neurolucida software we evaluated mean fiber area, quadriceps cross-sectional area and minimal Feret's diameter. We also analyzed the diaphragm NMJs to study their structure and maturation, as previously reported (NT-1654 WT *n* = 5, PBS WT *n* = 6).

### Statistics

Data are shown as mean ± standard error of mean (SEM). For the histological and molecular analysis, statistically significant differences among means were determined by unpaired *T*-test.

For the body weight, we used two-way ANOVA repeated measures, followed by post hoc Bonferroni; moreover, according to El-Khodor et al. (2008), data were also represented as a Kaplan–Meier plot, defined as the time from one initiating event (i.e., the birth, P0) to a terminating event: the latter one is considered “the postnatal day when (1) the animal lagged in body weight by two standard deviations from the established normal average body weight gain of the WT controls; and (2) the body weight remained two standard deviations below the average on each subsequent days” until the last observation (P10).

For the behavioral tests expressed as score or with data not normally distributed (i.e., righting reflex, tail suspension test, hindlimb suspension test-score, negative geotaxis), we applied contingency table analysis (Fisher exact test), whereas for the hindlimb suspension test-time (characterized by Gaussian distribution) we used one-way ANOVA repeated measures,

Finally, for evaluating the animal lifespan, we employed Kaplan–Meier survival curve that was generated with SPSS v.19 (IBM, Milan, Italy).

Differences were considered to be significant when *P* < 0.05. Statistical analysis was performed using GraphPad Prism 6.0 software (GraphPad Software, San Diego, CA) and SPSS v.19 (IBM). Experimenters were always blinded for the genotype and the treatment of the mice.

## Results

### Analysis of muscular/NMJ morphology and agrin expression in P10 WT and SMA mice

**Figure 1:**
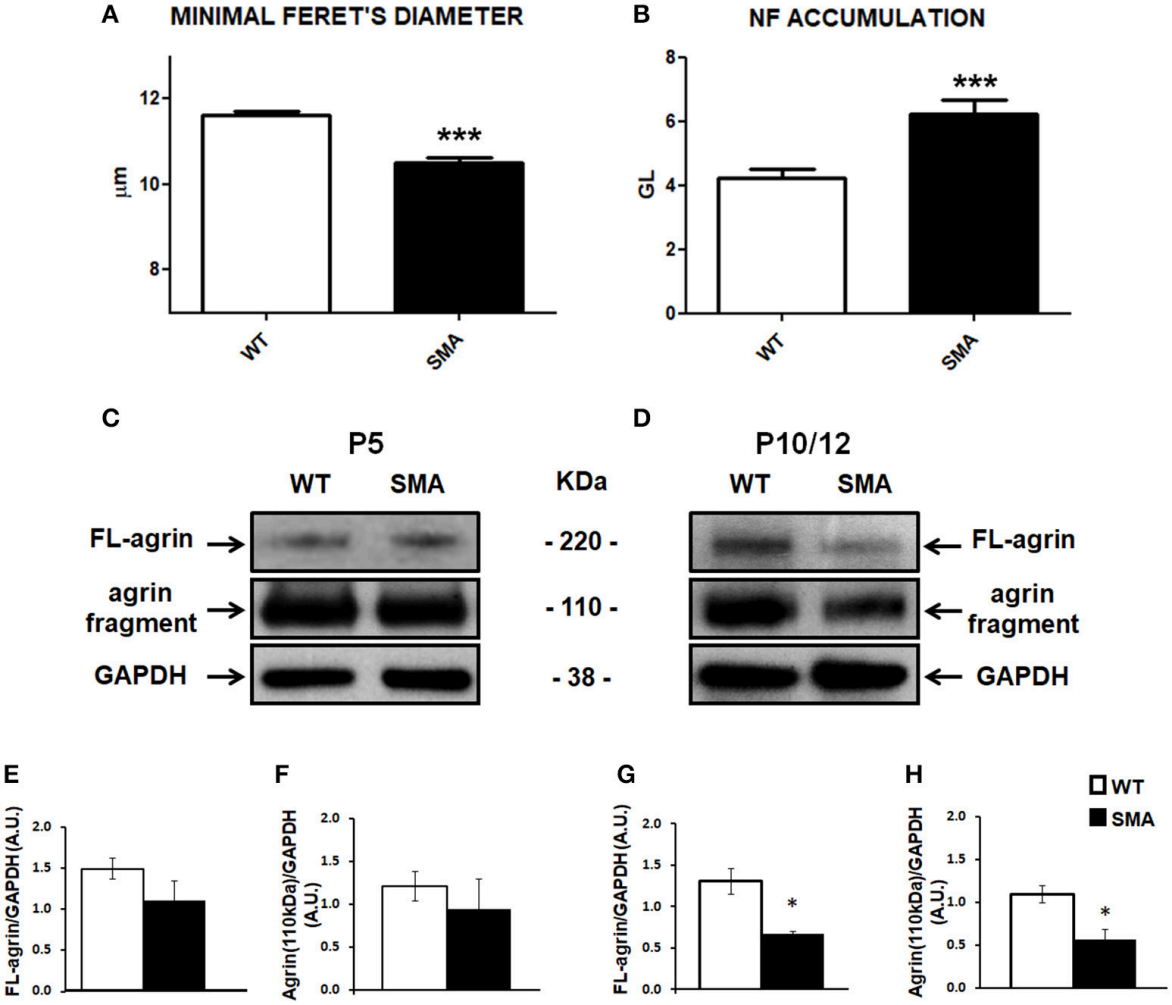
Analysis of muscular/NMJ morphology and agrin expression levels in the quadriceps of SMA mice. **(A)** Minimal Feret's diameter measured in P10 WT and SMA quadriceps. **(B)** Accumulation of neurofilament quantified by Imaris software and expressed as gray level. **(C,D)** Representative Western blots displaying full length (FL) agrin protein and the proteolytic fragment of 110 kDa in quadriceps of WT and SMA mice at P5 **(C)** and at P10 **(D)**. Graphs below each image report the quantification of FL-agrin/GAPDH protein levels at P5 **(E)** and P10 **(G)**, and the 110 kDa agrin fragment at P5 **(F)** and P10 **(H)**. Unpaired *T*-test, ^*^P < 0.05, ^***^P < 0.001.

NMJ and muscular fiber morphology are dramatically altered in SMA (Boido and Vercelli, 2016). We previously reported significant differences between SMA and WT pups in terms of mean fiber area and fiber type composition of quadriceps, an early affected muscle (Valsecchi et al., 2015). Now we have further highlighted such differences by measuring the minimal Feret's diameter, that is the most widely recommended parameter to determine the muscle fiber size (Dubache-Powell, 2008): after hematoxylin/eosin staining, WT and SMA fibers were analyzed by Neurolucida software, and respectively measured 11.61 ± 0.10 μm and 10.51 ± 0.10 μm (unpaired *T*-Test, *P* < 0.001; Figure 1A).

Moreover also NMJs are strongly affected in SMA, showing reduced endplate area and increased number of multi-innervated and denervated NMJs (Valsecchi et al., 2015). SMA endplates are also characterized by abnormal neurofilament (NF) accumulation, that we have now quantified, by using Imaris software: we detected a significant increase of NF in the SMA mice [6.24 ± 0.42 (expressed as “gray levels”, GL, as suggested by the software supplier)] compared to WT (4.22 ± 0.27) (unpaired *T*-test: *P* < 0.001; Figure 1B).

To evaluate the contribution of agrin in determining the described alterations, we quantified its expression level in the quadriceps of SMA mice at P5 and P10 by WB analysis. In particular, we measured the intensity of the full length protein (FL-agrin) that appeared approximately at 220 kDa: at P5 we observed a slight decrease of FL-agrin expression in SMA quadriceps compared to age-matched WT mice (Figures 1C,E; not significant), whereas at P10 a 50% reduction was evident in quadriceps of SMA mice in comparison with controls (*P* < 0.05; Figures 1D,G).

We also measured the intensity of the proteolytic fragment of agrin of 110 kDa in both SMA and WT mice at P5 and P10. The trend was similar to the FL-agrin with a 50% reduction of the proteolytic fragment in quadriceps of P10 (but not P5) SMNΔ7 mice, compared to age matched WT, indicating that the decrease in FL agrin was not due to increased cleavage of the protein (Figures 1C,D,F,H).

### Effects of treatment on general conditions and survival

To test whether the lowering of agrin in SMA may result in functional changes, we decided to administer NT-1654 to SMA pups with the hope to stabilize the NMJ and to improve the crosstalk between muscle fibers and MNs.

**Figure 2:**
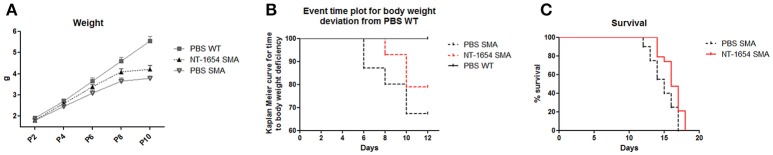
NT-1654 improves overall state of wellbeing. **(A)** Body weight evaluation during time (PBS WT *n* = 20, NT-1654 SMA *n* = 27, PBS SMA *n* = 15). **(B)** Event time plot for body weight deviation of PBS SMA, NT-1654 SMA and PBS WT (Kaplan–Meier curve, Log-rank Mantel Cox test, *P* < 0.05). **(C)** Kaplan–Meier survival curve shows that NT-1654 can significantly extends SMA lifespan (PBS SMA *n* = 20, NT-1654 SMA *n* = 19; Log-rank statistical test, *P* < 0.05).

Compared to the body weight of WT pups that constantly increased from P0 to P10, the SMA weight, after an initial moderate increase, at P8 reached a plateau and then started to decrease until death (data not shown). Administration of NT-1654 resulted in a slight weight increase in SMA mice (at P10 PBS SMA 3.78 ± 0.12 g vs. NT-1654 SMA 4.21 ± 0.19 g) (Figure 2A). However as suggested by El-Khodor et al. (2008), this type of representation (i.e., mean body weight for each group) does not provide the proper onset of SMA worsening as well as the proportion of the mice showing symptoms. Therefore, we converted the previous graph in a Kaplan–Meier plot: in this way we could observe that PBS SMA mice show an anticipated body weight reduction compared to NT-1654 SMA mice (Kaplan-Meier curve, Log-rank Mantel Cox test, *P* < 0.05), although both groups were clearly worse than PBS WT (considered the control mice; Figure 2B).

Additionally, in a separate set of experiments, we analyzed the NT-1654 efficacy in extending SMA lifespan. NT-1654 SMA mice showed an evident shift to the right in the survival (Kaplan-Meier curve, Log-rank statistical test, *P* < 0.05; Figure 2C); additionally, the mean day of death was 14.85 ± 0.39 for PBS SMA and 16.21 ± 0.33 for NT-1654 SMA (unpaired *T*-test, *P* < 0.01).

### Behavioral tests

**Figure 3:**
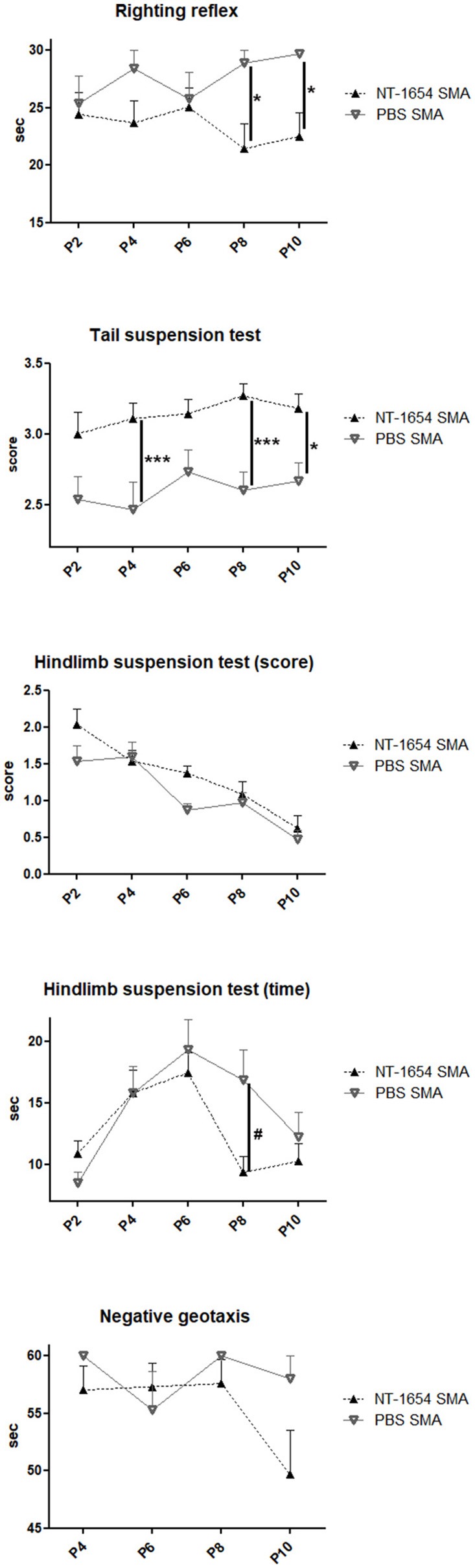
Behavioral tests. Righting reflex, tail suspension test, hindlimb suspension test (score and time) and negative geotaxis have been used to evaluate the motor performance of treated and untreated SMA mice (NT-1654 SMA *n* = 27, PBS SMA *n* = 15); Fisher exact test ^*^P < 0.05, ^***^P < 0.001; One-way ANOVA repeated measures, ^#^*P* < 0.05.

Starting from P2 until death, the motor performance of treated and untreated SMA mice was evaluated with a battery of behavioral tests (Figure 3). In the righting reflex test, NT-1654 administration resulted in a significant improvement in SMA mice compared to SMA PBS, starting from P8 (Fisher exact test: at P8, *P* < 0.05; at P10, *P* < 0.05). In the tail suspension test, starting from P4, SMA mice treated with NT-1654 showed a significant improvement compared to PBS-treated mice (Fisher exact test: at P4, *P* < 0.001; at P8, *P* < 0.001; at P10, *P* < 0.05). In the hindlimb suspension test, we considered two parameters, the score and the time. The scores registered were not significantly different between treated and untreated mice (analyzed by Fisher exact test); however, as concerns the latency to fall (time), at P8 NT-1654 SMA mice showed higher resistance compared to controls (One-way ANOVA repeated measures: *P* < 0.05). Finally, we also assessed motor coordination and vestibular sensitivity by measuring negative geotaxis in P4 to P10 mice. SMA animals (both treated and untreated) showed difficulties in completing the test for the entire observation period. However, even though lacking statistical significance, the performance of NT-1654 SMA showed better results (49.68 ± 3.85 s) than PBS SMA mice (58.00 ± 2.00 s).

### Muscle fiber analysis

**Figure 4:**
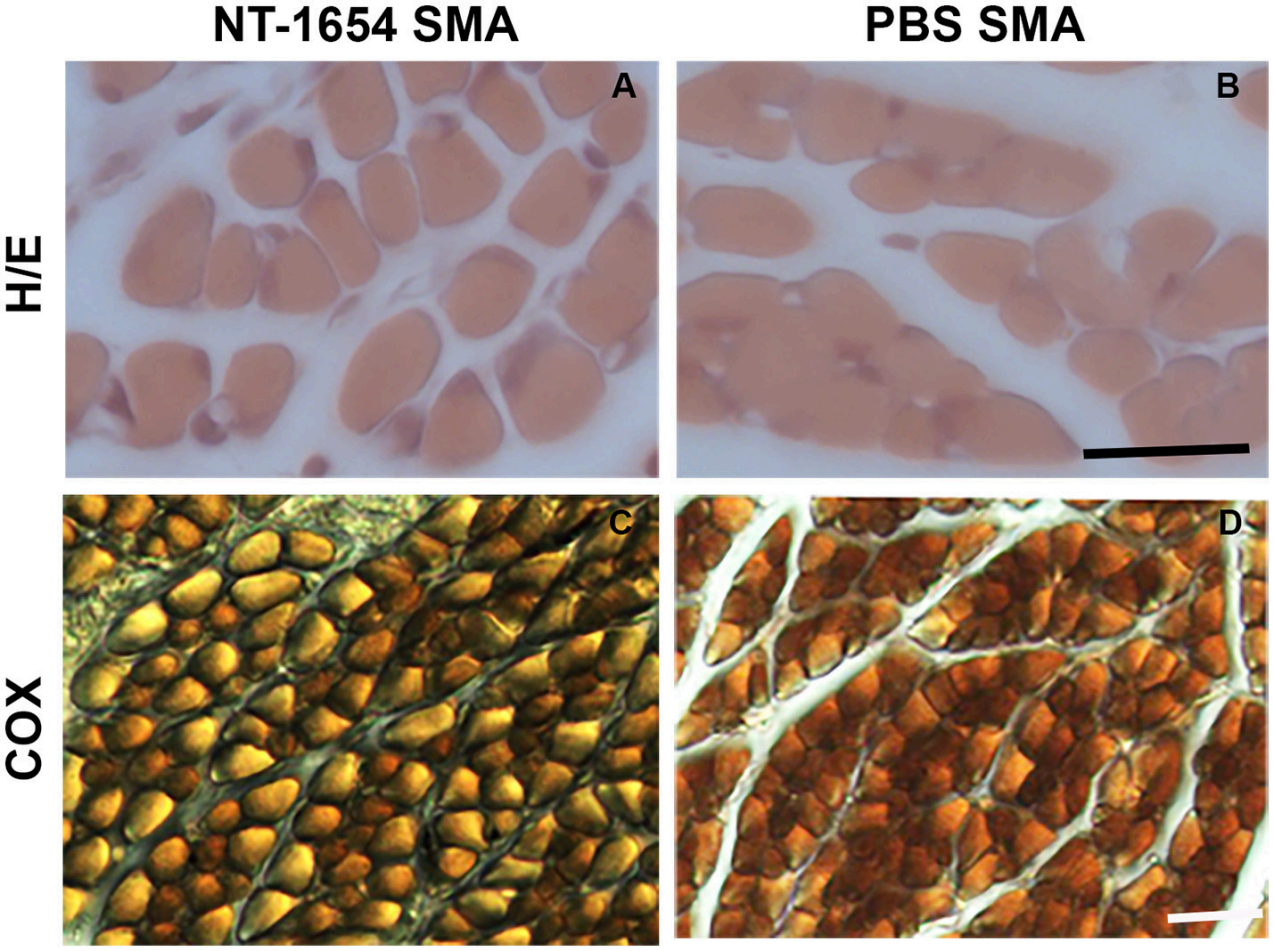
Analysis of quadriceps muscles. **(A,B)** The morphology of the muscles is highlighted by hematoxylin/eosin staining: NT-1654 administration increases the fiber size of SMA mice (NT-1654 SMA *n* = 6, PBS SMA *n* = 5). **(C,D)** COX reaction was used to classify the fiber types: NT-1654 treatment changed the fiber type distribution in SMA mice (NT-1654 SMA *n* = 4, PBS SMA *n* = 3). Scale bar = 25 μm in **(A,B)**, 35 μm in **(C,D)**.

After hematoxylin/eosin staining (Figures 4A,B), we analyzed the morphology of the muscle samples by Neurolucida software. We evaluated the mean fiber area, the overall muscle (quadriceps) cross-sectional area, the minimal Feret's diameter and the mean number of muscle fibers in treated and untreated SMA pups: we observed statistically significant differences in the mean fiber area and in the minimal Feret's diameter (NT-1654 SMA vs. PBS SMA; unpaired *T*-test, *P* < 0.001). The other parameters, though showing a positive trend to increase in treated mice, did not reach statistically significant differences (data are summarized in Table 1). These results suggest that the treatment prevents the decrease in size of muscle fibers, without affecting their number.

**Table 1:** Morphological analysis of quadriceps fibers.

	**NT-1654 SMA**	**PBS SMA**
Mean fiber area	141.18 ± 2.39^#^	127.39 ± 2.09
Quadriceps area	3, 083, 940 ± 230, 939	2, 595, 276 ± 61, 307
Min Feret's diam	11.20 ± 0.10^#^	10.51 ± 0.10
Fiber number	7, 602.53 ± 860.16	7, 206.65 ± 1, 361.71
% Fiber I	81.86 ± 1.95	88.33 ± 0.33
% Fiber II	18.14 ± 1.95	11.67 ± 0.33

The mean fiber area (μm^2^), the overall muscle cross-sectional area (μm^2^), the minimal Feret's diameter (μm), the mean number of muscle fibers and the fiber distribution were analyzed in treated and untreated SMA. Data are reported as mean ± SEM, statistical analysis was performed with unpaired T-test (NT-1654 SMA n = 6, PBS SMA n = 5). Mean fiber area and minimal Feret's diameter: NT-1654 SMA vs. PBS SMA

# *P < 0.001*.

We also employed COX reaction to classify the fiber types and to evaluate mitochondrial activity (Figures 4C,D). Treatment partially affected the fiber type distribution in SMA mice (differences not statistically significant): type I fibers were 81.86% ± 1.95 in NT-1654 SMA and 88.33% ± 0.33 in PBS SMA (consequently type II fibers were 18.14% ± 1.95 and 11.67% ± 0.33 respectively; see Table 1). Therefore, NT-1654 administration seems to bring back the fiber type distribution close to WT mice [type I and type II respectively 82% ± 1.82 and 18% ± 1.82, as reported in Wyatt and Keirstead (2010)].

Finally, we quantified the percentage of denervated endplates, studying the quadriceps NMJs immunoreacted by α-bungarotoxin and neurofilament. NT-1654 administration in SMA mice decreased the percentage of denervated endplates (4.33% ± 1.36) compared to untreated pups (5.54% ± 1.94).

### Neuromuscular junction analysis

**Figure 5:**
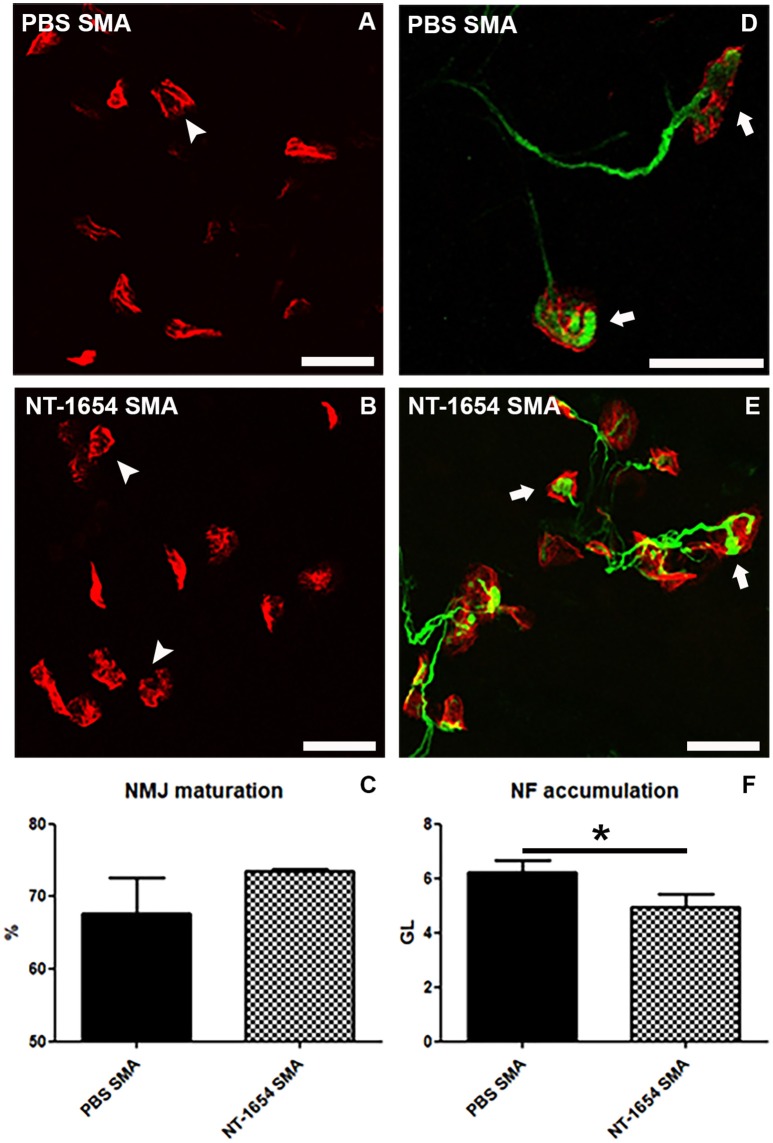
Analysis of diaphragm NMJs. **(A–C)** Evaluation of NMJ maturation by BTX-immunostaining: NT-1654 injection partially stimulates NMJ maturation in treated SMA **(B)** compared to PBS SMA **(A)**, as quantified in **(C)**. Mature endplates are indicated by arrowheads. **(D–F)** Double immunostaining against BTX (red) and NF (green) is employed for analyzing NF accumulation into the plaques: NT-1654 SMA **(E)** display a significantly lower number of engulfed NMJs (arrows) compared to PBS SMA **(D)**, as also quantified in **(F)**. Unpaired *T*-test, ^*^P < 0.05. Scale bar = 25 μm in **(A,B,D,E)**.

We performed a detailed morphological analysis of NMJs in the diaphragm. Even though this muscle is less severely affected than others in SMA, it allows to analyze all its NMJs in a whole-mount preparation. First of all, we classified the NMJs as mature (i.e., showing perforations) and immature (no perforations) (Figures 5A–C) (Bolliger et al., 2010). According to the previous observations on the quadriceps, the NT-1654 administration seems partially effective on the SMA diaphragm NMJs: the treatment increased the percentage of mature NMJs in NT-1654 SMA (74%) compared to PBS SMA (68%), although without reaching a statistically significant difference (Figure 5C).

Finally, by using Imaris software, we evaluated the NF accumulation in the NMJs (Figures 5D–F), since it is a SMA hallmark representing an engulfment at the NMJ level. We detected a significant reduction of NF in the treated SMA mice (NT-1654 SMA 4.93 ± 0.51 vs. PBS SMA 6.24 ± 0.42; unpaired *T*-test, *P* < 0.05).

### Spinal motor neurons stereological counts

We performed stereological counts in order to evaluate whether NT-1654 could also influence MN survival. By StereoInvestigator software, we counted L1-L5 lumbar MNs: only a slight tendency to higher MN numbers was evident in treated SMA mice vs. untreated ones (NT-1654 SMA 8,412.13 ± 964.21 vs. PBS SMA 7,351.84 ± 1,169.34), without reaching a statistically significant difference.

### NT-1654 administration to WT animals

To better understand if the observed histological/behavioral improvements in SMA animals were actually related to the therapeutic effect of NT-1654, rather than to a general effect on NMJ/muscle development, we administered the compound to a group of WT pups. We evaluated the body weight, the quadriceps morphology (mean fiber area, overall muscle cross-sectional area, minimal Feret's diameter, mean number of muscle fibers), the diaphragm NMJ maturation, and the behavioral performance.

**Figure 6:**
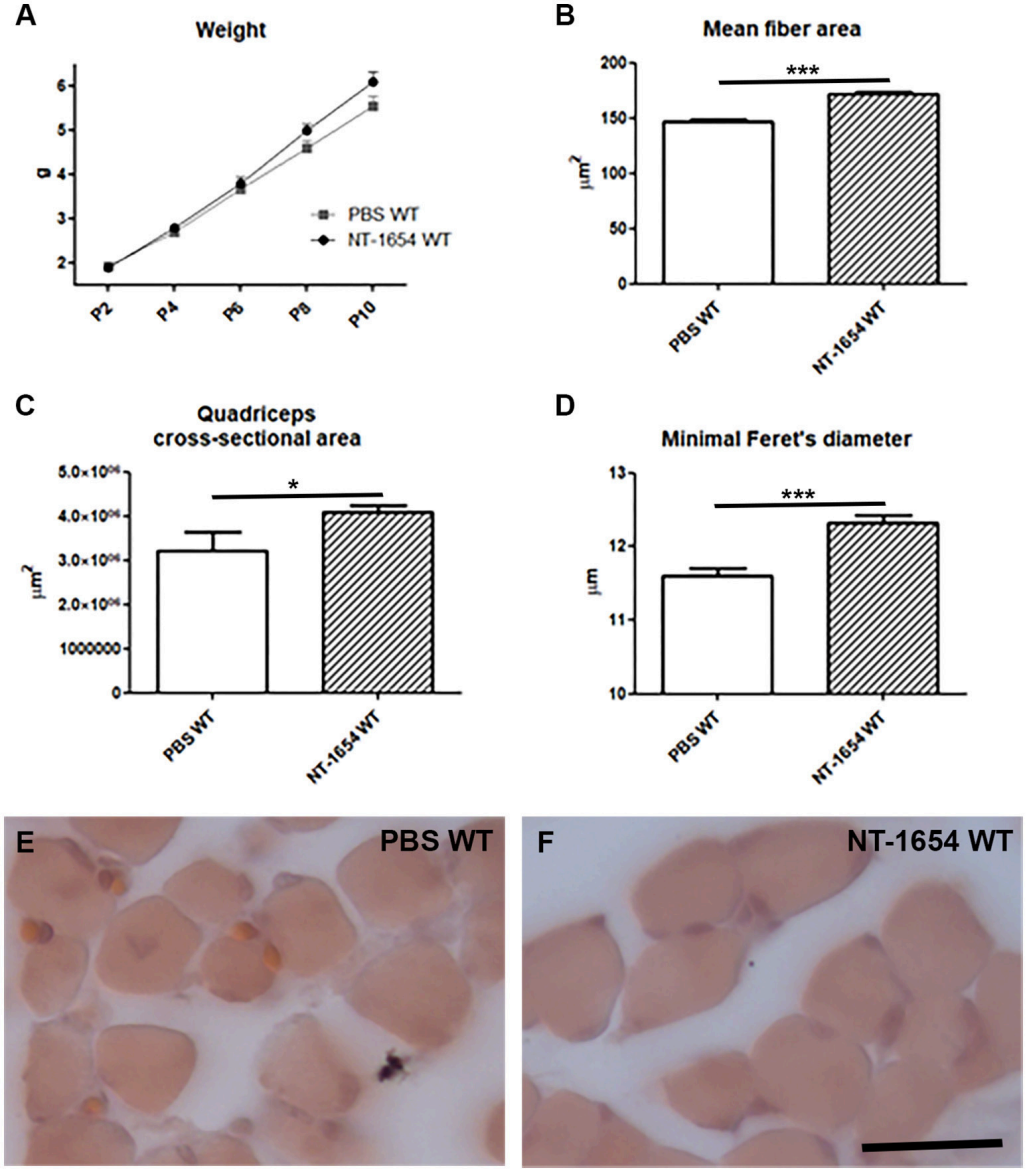
NT-1654 effects on P10 WT pups. **(A)** Body weight evaluation during time (PBS WT *n* = 20, NT-1654 WT *n* = 32). **(B–F)** Evaluation of mean fiber area **(B)**, quadriceps cross-sectional area **(C)**, and minimal Feret's diameter **(D)** in hematoxylin/eosin stained WT quadriceps **(E,F)** (NT-1654 WT mice *n* = 8, PBS WT *n* = 6; unpaired *T*-test, ^*^P < 0.05, ^***^P < 0.001). Scale bar = 25 μm in **(E,F)**.

Although the body weight was not considerably affected by the treatment (NT-1654 WT *n* = 32, PBS WT *n* = 20; Figure 6A), we observed a significant increase in the muscle fiber size (mean fiber area, quadriceps cross-sectional area, minimal Feret's diameter; NT-1654 WT mice *n* = 8, PBS WT *n* = 6), as shown in Figures 6B–F. On the contrary, neither NMJ maturation (NT-1654 WT *n* = 5, PBS WT *n* = 6) nor motor performance (NT-1654 WT *n* = 32, PBS WT *n* = 20) were affected by NT-1654 administration (only in the tail suspension test the treatment seemed to minimally accelerate the spreading of the hindlimbs at P2; data not shown). No adverse effects have been observed in WT mice after NT-1654 administration.

## Discussion

Here we evaluated the role of agrin/neurotrypsin system in a murine model of SMA: we observed that interference with this system via injection of the neurotrypsin-resistant agrin fragment NT-1645 exerted beneficial effects on muscle trophism and NMJ maturation, restoring the crosstalk between muscles and MNs and improving motor performance and extending survival.

### SMA and NMJ alterations

SMA is characterized by MN degeneration, due to deletion or mutation of the telomeric SMN1 gene and to the insufficient efficiency of SMN2 gene: this determines in the affected patients muscular atrophy, paralysis and reduced lifespan, according to the disease severity (Lorson et al., 2010). The SMA pathogenesis has been extensively investigated, but a number of disease mechanisms are still not fully understood. The SMN complex, composed of the SMN protein and 7 additional proteins (Gemin2-8), plays a pivotal role in the assembly and biogenesis of snRNPs, and in pre-mRNA splicing. SMN is reported to be also involved in the transport of axonal mRNAs in MNs, for example functioning as a molecular chaperone for β-actin messenger RNA localization in axons (Kolb et al., 2007; Tsai, 2012). Additionally, SMN is implicated in the stabilization and maturation of the NMJ: indeed, human SMA patients and experimental mouse models display consistent electrophysiological changes accompanied by a disruption of NMJ connectivity and denervation of skeletal muscles (Murray et al., 2010).

NMJs are specialized synapses in the peripheral nervous system, which allow the transmission between the motor nerve terminal and skeletal muscle fibers. As reviewed by Witzemann (2006), in physiological conditions, starting from embryonic days (E) 12–14 in mouse, AChRs are prepatterned at sites where the outgrowing MNs will form synaptic contacts; the mature endplates become stabilized at E17. During postnatal development, further structural changes occur and AChR aggregates are rearranged, acquiring the characteristically shaped “pretzel like” pattern.

Some molecular mechanisms are probably altered at the NJMs of SMA patients and consequently one of the earliest defects of SMA is found distal to the α-MNs, at the endplate level. As reviewed by Goulet et al. (2013), many studies suggest that in SMA the distribution of NMJs establishes at first as in controls, then several perturbations and the failure of synapse maturation lead to their loss, muscular denervation and, finally, symptom onset. Indeed, some authors have proposed a delayed development of SMA NMJs: AChR plaques appear immature in morphology, motor nerve terminal fails to produce the normal number and distribution of synaptic vesicles, many fibers retain polyneuronal innervation far longer than expected. Since these characteristics are typical of younger stages of neuromuscular development, the hypothesis is that SMA pathology delays the correct development (Kong et al., 2009; Lee et al., 2011). One possible reason explaining such abnormalities could be related to the role of the SMN protein in the generation of the pre-mRNA splicing machinery and mRNA biogenesis: indeed the lack of SMN may affect the production of incorrectly spliced transcripts involved in NMJ establishment/maintenance.

The muscle-nerve crosstalk is fundamental in regulating the formation of synapses, in association with an intricate interplay of many signaling molecules, including agrin, Lrp4, MuSK, Dok7 and rapsyn (Witzemann, 2006; Bolliger et al., 2010; Punga and Ruegg, 2012). Neurotrypsin is a member of the serine protease family S1A, predominantly found in the brain, the kidney, and the lung. Recent studies identified the proteoglycan agrin as the so far unique target of neurotrypsin. Agrin is a large heparan sulfate proteoglycan, expressed in neuronal as well as non-neuronal tissues. Neurotrypsin cleaves agrin at two sites (α and β) (Reif et al., 2008): the region of agrin corresponding to the 90-kDa fragment was shown to interact specifically with α-dystroglycan, integrins, and heparin that are expressed by neurons or astroglia enclosing the synapse; similarly, the 22-kDa agrin fragment was shown to be required to induce postsynaptic differentiation via its binding to Lrp4 (Stephan et al., 2008; Zong et al., 2012). Therefore, agrin is the synaptic organizer responsible for NMJ development, including the insertion of a nerve terminal, the clustering of AChRs and the maturation of NMJs (Bolliger et al., 2010).

SMA pups display at the NMJ reduced synaptic vesicle density, altered transmission, NF accumulation, immature and denervated endplates. The loss of innervation would result from defects in synapse maintenance, and also NF accumulation would be independent from NMJ vulnerability to denervation (Ling et al., 2010). Such neuropathological alterations could be based on an impairment in neurotrypsin/agrin system, as supported by the literature (Zhang et al., 2013; Kim et al., 2017) and our molecular observations: it is possible that MNs, mainly affected by the lack of SMN, stop producing agrin (Zhang et al., 2013), but muscles can partially extend its production.

### Therapeutic potential of agrin biological NT-1654

Agrin biological NT-1654 consists in a 44 kDa fragment of the C-terminal end of murine agrin: it is an engineered fragment, neurotrypsin-resistant, highly soluble and retaining the AChR clustering activity. Agrin NT-1654 can induce AChR clustering when administered *in vitro* to C2C12 myotubes, and *in vivo* to C57/Bl6 denervated mice (Hettwer et al., 2014).

In SMA mice, the synaptic pathology at the NMJ is evident and appears at early symptomatic stage; sometimes also morphologically intact NMJs can display functional alterations (Murray et al., 2010). The lack of SMN protein impairs the post-natal development of NMJs, affecting the maturation of AChR clusters into pretzel-like profiles and leading to intermittent neurotransmission failures (Kariya et al., 2008). Therefore, our therapeutic approach starting at P0 aimed to prevent these events.

In agreement with the literature (Ling et al., 2010; Dale et al., 2011), we observed that SMA NMJs display remarkable abnormalities, such as immaturity, reduced size, NF accumulation and denervation [present results, and (Valsecchi et al., 2015)]. Similar defects have been also observed in a sarcopenia murine model (neurotrypsin-overexpressing SARCO mice), which is characterized by muscle loss similar to the loss observed at late age: indeed these animals showed NMJ fragmentation, defective innervation and incomplete maturation. NT-1654 injections almost completely restored the normal conditions, supporting the rebuilding of the disassembled endplates (Hettwer et al., 2014). Here the treatment with NT-1654 induced positive effects on NMJ morphology, stimulating their maturation, probably by acting both on AChR clustering and on agrin/Lrp4/MuSK signaling. Additionally, the accumulation of NF, a typical SMA hallmark, was significantly reduced after treatment. All these aspects can contribute to reduce the impaired synaptic functions related to the disease (for an extensive review see, Boido and Vercelli, 2016).

Due to the improved NMJ functionality/maturation, we also observed a higher trophism of the muscles, probably due to better innervation. Indeed, the mean fiber area was significantly larger in NT-1654 SMA than in PBS controls, and also the other analyzed parameters (muscle cross-sectional area and mean number of muscle fibers) showed a similar positive trend.

Also the fiber composition was influenced by the treatment, which restored the percentage of fiber type I (slow) and type II (fast) close to WT values (Valsecchi et al., 2015). As described by Dubowitz (1978), in case of SMA, type II fibers are the most affected and are characterized by an extensive atrophy, partially compensated by type I fibers. Other pathologies causing muscle wasting (such as Duchenne muscular dystrophy or cancer cachexia) show a similar fast-to-slow fiber type shift, with type II fibers preferentially affected (Ciciliot et al., 2013). NT-1654 administration was able to reverse the hybrid fibers (co-expressing several myosin heavy chain isoforms) to the normal segregation of type I and II fibers in SARCO mice, demonstrating that such treatment can positively influence the muscle fiber composition (Hettwer et al., 2014).

Additionally, the accumulation of NF, a typical SMA hallmark, was significantly reduced after treatment. It has been suggested that NF accumulation is due to a dysregulation of the axonal transport machinery (Kreutzer et al., 2012) leading to an impaired NMJ maturation and maintenance (Torres-Benito et al., 2012). Even though the NF engulfment is still matter of debate, our data support the hypothesis of a relationship between NF accumulation, axonal transport and endplate development.

Overall, these observations demonstrate that NT-1654 administration improves innervation, supports NMJ maturation and consequently influences the muscle trophism. However, the positive effects observed at the peripheral level only partially affect MN survival: a tendency in higher MN numbers was quite evident in treated SMA mice vs. untreated ones, even though it did not reach statistical significance.

Finally, the improvements observed at histological level were also positively correlated with behavioral data. Indeed, we show that NT-1654 administration results in a significant improvement in SMA mice in the righting reflex, the tail suspension test and, to a small extent, the hindlimb suspension test, indicating its positive effect on weakness and fatigue. Additionally, survival was slightly, but significantly, increased: we are aware that 1.5 days of extended lifespan could appear poor, but it must be related to the usually short SMA lifespan of around 14 days.

Other groups tried to rescue endplates and increase muscle size in SMA, but the achieved improvements of neuromuscular synaptic phenotypes often did not correlate with similar positive outcomes concerning survival, motor behavior or histological results (Paez-Colasante et al., 2013; Wen et al., 2013). Indeed the agrin expression modulation appears an effective target to positively influence the NMJ establishment/maintenance, as recently demonstrated by Kim et al. (2017): by generating SMNΔ7 pups expressing a chicken Z^+^Agrin cDNA driven by the motor neuron-specific Hb9 promoter (*Hb9:Z*^+^*Agrin*;*SMN2*;*SMN*Δ*7*;*Smn*^−/−^), the authors obtained results comparable to ours (i.e., muscular fiber and NMJ size increase, NF reduction and improved muscular innervation). Therefore, our pre-clinical work i) confirms the importance of targeting agrin to delay the SMA progression, and ii) identifies an effective compound (NT-1654) that could be potentially tested in human trials.

Finally, we noticed that NT-1654 is able to induce some positive effects also in WT mice, boosting the muscle size (without significant effects on NMJ maturation and innervation). These data suggest that NT-1654 can interfere with the muscular homeostasis in healthy subjects, confirming that agrin can also exert non-canonical functions, for example related to skeletal muscle plasticity/differentiation (Gros et al., 2014) and contractile apparatus maturation (Mis et al., 2013). Moreover, the administration of NT-1654 in WT animals has demonstrated that such treatment (i) does not negatively influence the correct murine development, (ii) does not induce particular side effects in pups and consequently (iii) can be considered safe. Moreover, as shown in another study, NT-1654 does not induce immune response, nor anti-agrin antibody production (Li et al., 2017).

One of the major symptoms of SMA and other diseases showing muscle atrophy consists in the loss of muscle strength, which seriously impairs motor behavior of patients (Merlini et al., 2004). Targeting agrin might represent a good strategy to prevent muscle atrophy. Indeed, we can suppose that the beneficial effects obtained by NT-1654 administration in our SMAII experimental model could be greater in the milder forms of SMA (e.g., SMAIII), where the pathological cascade affecting MNs, NMJs and muscles occurs more slowly, in this way allowing a more extended therapeutic window.

### Concluding remarks on NT-1654 mechanism of action

As demonstrated by the above-mentioned histological and behavioral observations, the administration of agrin biological NT-1654 exerted beneficial effects in SMA mice. Actually, we can only speculate about the mechanisms of action of such treatment, and the relationship between SMN and agrin.

What is currently known is that SMA pups display a number of neuropathological alterations ranging from delayed NMJ maturation to NF accumulation at NMJs both in experimental models (Ling et al., 2010) and in human patients (Martínez-Hernández et al., 2013): a possible explanation for such early abnormalities could be found in an impairment of neurotrypsin/agrin system, as supported by a few evidence in literature. In 2013, Zhang et al. (2013) demonstrated that SMA MNs lack neuronal (Z+) agrin mRNA at P1, until its complete disappearance at P3, in SMAΔ7 mice. Based on their observations, they suggest that SMA mice are capable of expressing and producing Z+ agrin at P1: however, a problem in the skipping of Z exons is responsible of the observed Z+ agrin protein deficiency, determining a consequent impairment in postsynaptic AChR clustering. Here, by WB analysis, we observed a significant decrease in the overall agrin expression in the quadriceps of P10 SMA (as suggested by the reduced expression of 110 kDa proteolytic fragment). It is plausible that SMAΔ7 mice suffer an initial decrease of Z+ agrin (Lefebvre et al., 1997; Kim et al., 2017), followed by a general reduction of the overall agrin.

Also referring to other pathologies (congenital myasthenia, distal myopathy) the lack/mutation of agrin has been associated to muscle weakness, atrophy and neurotransmission defects (Nicole et al., 2014). Therefore, the administration of NT-1654 to our SMA experimental model has probably compensated for the reduction of agrin. Similarly, transgenic expression of a miniaturized form of agrin (“mini-agrin”), in combination with anti-apoptosis treatment, provided benefit on the disease progression in a mouse model of congenital muscular dystrophy, stimulating stabilization of muscle fibers, muscle growth/strength and regeneration (Meinen et al., 2011). Moreover, also overexpressing Z+ agrin in MNs of Nova1/Nova2 double knockout mice (an experimental model that show paralysis and defective neuromuscular synapses) rescued AChR clustering, but without restoring paralysis: the authors suggested that there must be additional agrin-independent neuronal factors required for MN function (Ruggiu et al., 2009).

Concerning SMA, other explanations for the treatment success obtained with NT-1654 could be found in the counteraction of the defects due to SMN lack. As known from the literature, SMN deficiency affects the splicing machinery activity (Lorson et al., 1999). Zhang demonstrated that SMN deficiency causes transcriptome and proteome changes in MNs, affecting specific genes necessary/detrimental for NMJ development, including agrin (Zhang et al., 2013). Moreover, splicing defects could also determine alterations in the microtubule-based motor proteins (kinesin and dynein) and other interacting proteins, justifying the well-known alteration of cellular trafficking in SMA (Dale et al., 2011): notably, dynein can affect the expression and the clustering of agrin-induced AChRs, MuSK and Rapsyn; in addition defects in dynein can lead to impairment of NMJs, and seem to be involved in ALS neurodegeneration (Vilmont et al., 2016). Moreover, we cannot exclude that also the transport of agrin could be affected/reduced. Therefore, the NT-1654 administration could overcome such deficiency.

Among the alterations described in SMA, it has been also reported that SMN-deficient cells show a disorganized actin cytoskeleton, affecting cell motility and myoblast fusion/differentiation (Bricceno et al., 2014). Actin is also necessary for AChR clustering and stabilization at the synapse: agrin can trigger the association of a subset of membrane rafts enriched in AChR and MuSK to the actin cytoskeleton (Cartaud et al., 2011). Such defects in SMA could be partially restored by NT-1654 administration, explaining the positive effects observed. Moreover, many alterations in SMA type II seem to have an embryonic onset: this should partially justify some limitations of our therapy.

In conclusion, on the basis of our observations, agrin biological NT-1654 represents a good therapeutic candidate for SMA, counteracting agrin deficiency and SMN-induced defects: it should be exploited in combination with the emerging promising therapies aimed at increasing SMN expression. Indeed, despite the first encouraging results coming from gene therapy and ASO approaches, additional treatments are needed to complement the benefits of CNS-directed and SMN-dependent therapies (Bowerman et al., 2017), for the following reasons: (i) the progressive deterioration of the neuromuscular system may compromise the positive effects due SMN-dependent treatments; (ii) many patients with milder SMA forms do not meet the inclusion criteria requested for clinical trials and may not benefit from SMN-inducing treatments.

Moreover, potentially we expect similar results also for other neuromuscular disorders affecting the motor system, such as Charcot Marie Tooth syndrome, myasthenia gravis and congenital myasthenic syndromes.

## Author contributions

MB, SH, and AV: Conceived and designed the experiments; MB, ED, VV, and MT: Performed the experiments; MB, ED, VV, UA, and AV: Analyzed the data; MB, AV: Wrote the paper; MB, VV, UA, MR, SH, and AV: Review the paper.

### Conflict of interest statement

SH is employed by Neurotune AG. However, while contributing to experiments and writing of the manuscript, his status did not affect planning of experiments, analysis and discussion of data. All other authors not affiliated with Neurotune AG were not influenced by Neurotune AG or its employee. This does not alter the authors' adherence to Frontiers in Cellular Neuroscience policies on sharing data and materials.

## Abbreviations

AChRs: acetylcholine receptors
BTX: α-bungarotoxin
COX: Cytochrome c Oxidase
MN: motor neuron
NF: neurofilament
NMJ: neuromuscular junction
PBS: phosphate buffer solution
SEM: standard error of mean
SMA: spinal muscular atrophy
SMN: survival motor neuron
snRNP: small nuclear ribonucleoprotein.
